# Revisiting adaptations of neotropical katydids (Orthoptera: Tettigoniidae) to gleaning bat predation

**DOI:** 10.1080/23766808.2016.1272314

**Published:** 2017-01-24

**Authors:** Hannah ter Hofstede, Silke Voigt-Heucke, Alexander Lang, Heinrich Römer, Rachel Page, Paul Faure, Dina Dechmann

**Affiliations:** ^a^ Department of Biological Sciences, Dartmouth College, Hanover, NH, USA; ^b^ Department of Biology, Chemistry, and Pharmacy, Freie Universität Berlin, Berlin, Germany; ^c^ Zoology Institute, University of Graz, Graz, Austria; ^d^ Smithsonian Tropical Research Institute, Ancon, Panama; ^e^ Department of Psychology, Neuroscience & Behaviour, McMaster University, Hamilton, Canada; ^f^ Department of Biology, Universitat Konstanz, Konstanz, Germany

**Keywords:** anti-predator defenses, Chiroptera, eavesdropping, Panama, predator-prey arms race

## Abstract

All animals have defenses against predators, but assessing the effectiveness of such traits is challenging. Neotropical katydids (Orthoptera: Tettigoniidae) are an abundant, ubiquitous, and diverse group of large insects eaten by a variety of predators, including substrate-gleaning bats. Gleaning bats capture food from surfaces and usually use prey-generated sounds to detect and locate prey. A number of Neotropical katydid signaling traits, such as the emission of ultrasonic frequencies, substrate vibration communication, infrequent calling, and ultrasound-evoked song cessation are thought to have evolved as defenses against substrate-gleaning bats. We collected insect remains from hairy big-eared bat (*Micronycteris hirsuta*) roosts in Panama. We identified insect remains to order, species, or genus and quantified the proportion of prey with defenses against predatory bats based on defenses described in the literature. Most remains were from katydids and half of those were from species with documented defenses against substrate-gleaning bats. Many culled remains were from insects that do not emit mate-calling songs (e.g. beetles, dragonflies, cockroaches, and female katydids), indicating that eavesdropping on prey signals is not the only prey-finding strategy used by this bat. Our results show that substrate-gleaning bats can occasionally overcome katydid defenses.

## Introduction

Predation is one of the strongest agents of natural selection [1], and in response, prey has evolved a spectacular diversity of anti-predator defenses [2]; however, the efficacy of prey defenses is difficult to quantify in nature. In the Neotropics, katydids (Orthoptera: Tettigoniidae) are an abundant, ubiquitous, and diverse group of large insects that are eaten by a variety of predators including bats, monkeys, rodents, birds, lizards, and amphibians [3,4]. Katydids exhibit an assortment of morphological and behavioral traits considered to be anti-predator defenses [5].

**Figure 1.**
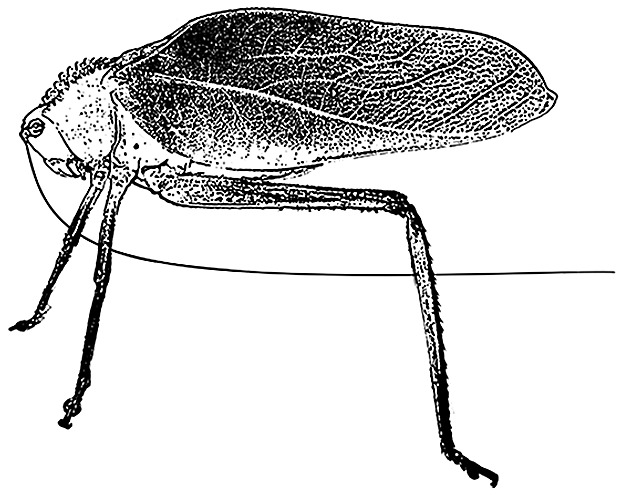
Lateral view of *Steirodon stalii* showing thoracic spines as thick protuberances.

Katydid morphological defenses include crypsis, mimicry, chemical defenses, spines, and a strong bite. Many katydids are visually cryptic, having broad green wings to blend in with vegetation, or other forms of coloration to blend in with bark or lichen. Likewise, there are a number of spectacular forms of mimicry found in katydids, such as wasp (*Scaphura* spp. and *Aganacris* spp.) and leaf (*Mimetica* spp., *Aegimia* spp., *Pycnopalpa bicordata*) mimics [6]. Some species have long, sharp spines on their legs or thorax (*e.g. Steirodon* spp., Figure 1) and a strong bite owing to their large mandibles (*e.g. Copiphora* spp.). Behavioral defenses include well-hidden daytime roosts [3,5], reduced activity during bright phases of the moon [7], singing from protected perches [8], and changes in acoustic behavior [4]. Male katydids use their wings to produce calling songs that attract females, and have acoustic defenses such as song cessation at the approach of a potential predator and the production of loud sounds when touched that might startle a predator [4]; however, the efficacy of these defenses in the wild remains unknown.

In the Neotropics, substrate-gleaning bats capture prey from surfaces and are known to exert top-down control on insect populations [9,10]. In addition, many gleaning bats are significant predators of katydids specifically [8,11–15, this study]. Many substrate-gleaning bats listen to the mate-calling songs of male katydids to detect and locate them as prey [8,16,17]. Thus, some acoustic characteristics of Neotropical katydid songs are believed to be specific adaptations against gleaning bat predation [3]. For example, sporadic/infrequent calling [8,18,19], the emission of high ultrasonic frequencies [20], and substrate vibration communication [8,20,21] have all been proposed as defensive forms of communication to evade detection by eavesdropping bats. Katydids have ultrasound-sensitive ears and some will cease singing in response to ultrasonic pulses and bat echolocation calls [22–25]. Cessation of singing can effectively thwart the gleaning attacks of temperate northern long-eared bats, *Myotis septentrionalis* [26], but the efficacy of such an acoustic defense has not been tested against Neotropical gleaning bats. For example, the Neotropical common big-eared bat *Micronycteris microtis*, a congener of our study species, uses biosonar to locate silent, motionless arthropods resting on leaf surfaces [27], suggesting that song cessation alone will not always prevent the predatory attacks of substrate-gleaning bats.

The goal of this study was to identify the katydid species eaten by the hairy big-eared bat *M. hirsuta*, a major gleaning bat predator, and to assess the proportion of katydid species with described anti-bat defenses in the diet. Previous studies have shown that *M. hirsuta* eats many katydids ([11]: 25% by number, [28]: 41% by number, 61.5% by weight); however, the relative number and sex of all katydid prey have never been reported. Detailed data about the dietary composition of bat predators can help address hypotheses about the efficacy of presumed katydid defenses.

### Methods

The study was conducted at the Smithsonian Tropical Research Institute (STRI) field station on Barro Colorado Island (BCI) in Panama (9°10′ N, 79°51′ W). The 15.6 km^2^ island is covered with moist, semi-deciduous tropical forest composed of both young and old stands (90–600 years old; [29]).

From November 2001 to January 2003, we collected arthropod remains from three hollow trees used as roosts by *M. hirsuta*. Bats were caught and identified to species when they emerged from the roost at night. We filmed the inside of one roost to confirm that the bats inside were responsible for dropping arthropod remains at night. This also demonstrated that bats frequently returned to the same roost to consume insects throughout the foraging period.

To collect culled arthropod parts, a plastic sheet was suspended off the ground inside the hollow tree roost to keep the prey remains dry and prevent them from being washed away by rainwater. From roost 1, collections were made 2–14 times/month covering all seasons except April and May (63 collections). Remains were also collected from two other *M. hirsuta* roosts (roost 2 collection dates: 27 September 2002, 21 November 2002 and 5 December 2002; roost 3 collection date: 29 November 2002). Cockroaches and ants were observed removing bat feces and, more rarely, insect remains, thus our samples likely underestimate the total number of prey items brought back to bat roosts. However, the purely chitinous remains that are important items for taxonomic classification, such as arthropod wings, legs, and ovipositors, were seldom removed.

Most insect parts were identified to order, and in case of katydids to the level of family, subfamily, genus, and species whenever possible using keys and information from [4,30–32]. For all insect remains, we report the total number of body parts found as a measure of the maximum number of individuals that were captured for each taxonomic group. For katydids, we additionally examined forewings to classify them as male (having characteristic sound producing structures) or female (lacking these structures). Some wings could not be classified in this way because they were missing the area of the wing with sound-producing structures and these were included as individuals of unknown sex in the count. We also identified each forewing as a left or right wing. By matching the left and right wings of the same sex, we were able to calculate the minimum number of individual katydids at each roost.

We conducted a literature search to document defenses of Neotropical katydids believed to be effective against predatory bats. These defenses were noted for all species of katydid from Panama and are reported here for those species found as remains in bat roosts (Table 1). A number of defenses are common to all Neotropical katydids and are not listed in Table 1: regurgitation of crop fluid (thought to be distasteful to predators; [3,5]), holding tightly to a substrate when grabbed [3], kicking [5], and autotomy of the hind legs [5]. Only defenses that vary by katydid species were documented, including large mandibles with a strong bite, large cuticular spines, defensive startle sounds, calling from protected perches, infrequent signaling, production of vibratory tremulation signals, and calling song cessation.

**Table 1.** Minimum number of individual katydids represented in remains collected from a *Micronycteris hirsuta* roost, and previously described anti-predator defenses specific to bats for each katydid species [sources in brackets].

Family and subfamily		Species	*N* total	*N* male	*N* female	*N* unknown sex	Anti-predator defenses specific to bats
Tettigoniidae		All	784	147	261	376	
	Conocephalinae	*Copiphora brevirostris*	23	9	6	8	Infrequent calling (< 1 call / min; [28]), communicates with tremulation signals [20,28], large mandibles and a strong bite [3], stops singing in response to bat echolocation calls [26]
		*Erioloides longipennis*	5	0	0	5	Unknown
		*Erioloides sp.*	2	0	0	2	Unknown
		*Neoconocephalus affinis*	1	0	0	1	Calls continuously from grassy areas where gleaning bats do not hunt [8]; does not stop singing in response to gleaning bat echolocation calls [24]
		*Subria sylvestris*	2	0	0	2	Unknown
		Unknown species	20	2	1	17	
	Phaneropterinae	*Anapolisia colossea*	2	1	1	0	Unknown
		*Anaulacomera sp.*	15	3	4	8	Unknown
		*Ectemna dumicola*	2	0	1	1	Unknown
		*Hyperphrona irregularis*	9	0	1	8	Unknown
		*Hyperphrona trimaculata*	3	0	0	3	Unknown
		*Itarissa sp.*	4	1	0	3	Unknown
		*Lamprophyllum bugabae*	2	0	1	1	Stops singing in response to bat echolocation calls (ter Hofstede, unpubl. data)
		*Lamprophyllum micans*	3	0	3	0	Unknown
		*Lamprophyllum sp.*	1	0	0	1	Unknown
		*Microcentrum sp.*	2	1	1	0	Unknown
		*Orophus tessellatus*	5	0	1	4	Unknown
		*Phylloptera dimidiata*	2	0	0	2	Unknown
		*Phylloptera festae*	2	0	1	1	Unknown
		*Steirodon stalii*	1	0	0	1	Dull but thick spines on thorax, sharp spines on hind legs, kicks hind legs when touched [3]
		*Viadana sp.*	1	0	0	1	Unknown
		Unknown species	22	3	1	18	
	Pseudophyllinae	*Acanthodis curvidens*	10	0	0	10	Infrequent calling (< 1 call / min; [28]), communicates with tremulation signals, large mandibles and a strong bite, kicks with spiny legs, anecdotally noted that the males continue to sing in the presence of bats; produces startling sound when touched [3]
		*Balboana tibialis*	5	2	1	2	Regular calling (approx. 4–5 calls / min; [24,28]), communicates with tremulation signals [3], stops calling in response to bat echolocation calls [24].
		*Bliastes punctifrons*	18	6	6	6	Unknown
		*Bliastes sp.*	5	0	0	5	Unknown
		*Brachyauchenus festae*	1	0	0	1	Unknown
		*Cocconotus wheeleri*	7	0	0	7	Regular calling (approx. 1 call / min), communicates with tremulation signals, anecdotally noted that the males continue to sing in the presence of bat-like sounds [28].
		*Cocconotus sp.*	16	4	3	9	Unknown
		*Docidocercus sp.*	163	28	70	65	Regular calling (5–6 calls / min; [24, 28]), communicates with tremulation signals [28], reduced activity during bright moonlight phases [7], does not stop calling in response to gleaning bat echolocation calls [24].
		*Idiarthron incurvum*	20	8	10	2	Unknown
		*Idiarthron majus*	3	0	0	3	Infrequent calling (< 1 call / min), communicate with tremulation signals [28]
		*Idiarthron sp.*	25	0	0	25	Unknown
		*Ischnomela gracilis*	8	0	1	7	Frequent caller (30–40 calls / min), stops calling in response to gleaning bat echolocation calls [24].
		*Ischnomela pulchripennis*	2	0	2	0	Has a continuous calling song, but calls from a spiny bromeliad for protection [28]
		*Melanonotus bradleyi*	27	5	10	12	Infrequent calling, communicates with tremulation signals [8]
		*Melanonotus sp.*	28	5	9	14	Unknown
		*Mimetica incisa*	7	1	0	6	Leaf mimic [4]
		*Mimetica viridifolia*	4	0	0	4	Leaf mimic [4]
		*Mimetica sp.*	1	0	0	1	Leaf mimic [4]
		*Parascopioricus lancifolius*	11	1	4	6	Unknown
		*Pristonotus tuberosus*	8	0	1	7	Infrequent calling (ca. 1 call / min), communicates with tremulation signals, males produce a startling sound when touched [28]
		*Thamnobates subfalcata*	6	1	2	3	Unknown
		*Xestoptera cornea*	130	64	53	13	Regular calling (approx. 1 call / min), communicates with tremulation signals, males produce a startling sound when touched [28]
		Unknown species	150	2	67	81	

## Results

**Figure 2.**
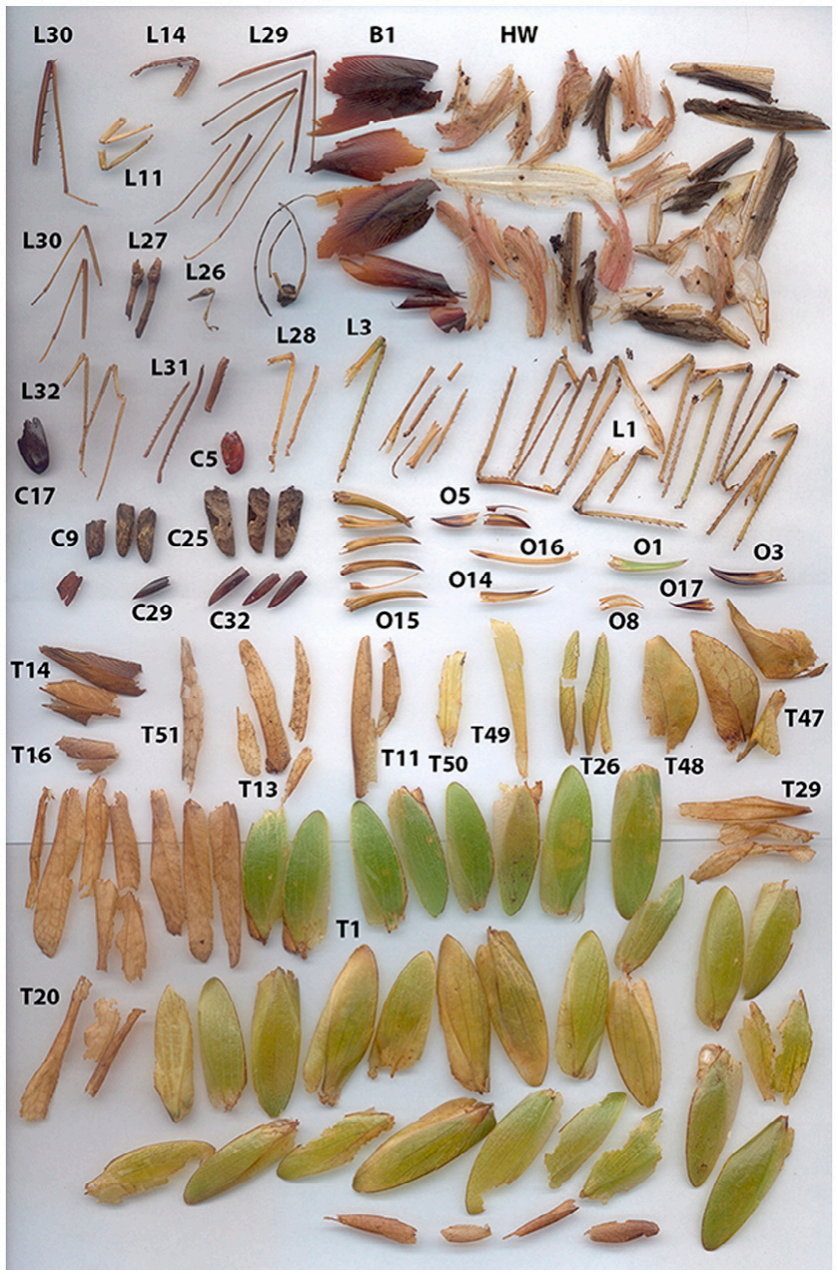
Sample of culled katydid remains with identification numbers collected from a single *Micronycteris hirsuta* roost on one day. B: Blattodea wings, C: Coleoptera wings, T: Tettigoniidae (katydid) forewings, HW: hindwings, L: legs, O: ovipositors.

At three *M. hirsuta* roosts, we found a total of 1,931 culled arthropod parts, which were mostly insect wings, ovipositors, and legs (Figure 2). Out of all the remains, 109 were Blattodea (6%), 296 were Coleoptera (15%), 1,443 were Orthoptera (75%), 25 were Odonata (1%), and 58 could not be identified to order (3%). Of the Orthoptera, 72 were from the family Gryllidae (crickets), 1,341 were from the family Tettigoniidae (katydids), and 30 could not be identified to family. Therefore, by far the most abundant group of the insect remains were the katydids, comprising 69% of all remains at the roosts.

Based on wing morphology, we calculated the minimum number of katydid individuals as 784 (Table 1; see methods). In agreement with Belwood [28], the majority of katydids eaten by *M. hirsuta* were from the subfamily Pseudophyllinae (655, or 83% of katydids), with fewer individuals from the subfamilies Phaneropterinae (76, 10%) and Conocephalinae (53, 7%). More than half of the katydids (486, 62%) could be identified to genus or species (Table 1). Six identified genera made up 56% of all katydid individuals collected from bat roosts: *Docidocercus*, *Xestoptera*, *Idiarthron*, *Copiphora*, *Melanonotus*, and *Cocconotus*. With the exception of *Copiphora*, these are all genera from the subfamily Pseudophyllinae.

Based on data from Orthoptera Species File Online [32], there are approximately 130 katydid species in Panama. We found literature supporting defensive behavior for 38 of these species. For the katydid remains found in the bat roosts, we were able to classify katydids into 42 species or species groups, 12 of which had documented defensive behavior against bats (Table 1).

Of the identified katydids, roughly 50% (390/784) came from 14 species with documented anti-predator defenses (Table 1). Of these 14 species, two possess large mandibles with a strong bite, two have prominent cuticular spines, three produce startle sounds when touched, two sing from protected perches, five sing sporadically, nine use tremulations for substrate-borne vibratory communication, and four exhibit song cessation in response to playbacks of bat echolocation calls. We could identify the sex of the katydids for 52% of the individuals (408/784). Of those for which the sex could be identified, 36% were males and 64% were females. Similar percentages of males and females were also found for individual species with large sample sizes (e.g. *Docidocercus* sp. and *Xestoptera cornea*; Table 1).

## Discussion

Our results show that the effectiveness of many proposed defenses of katydids against bats may not be as effective as originally suggested in the literature. Given the high proportion of female katydids and silent prey in the remains, our results also suggest that *M. hirsuta* use sensory cues other than prey-generated acoustic signals to find their prey. Although we cannot quantify the number of prey taken relative to their availability or the reduction in predation provided by specific antipredator defenses, our data nevertheless show that tropical substrate-gleaning bats at least occasionally overcome katydid antipredator defenses. The detailed level of prey identification in our study provides an opportunity to assess specific predator-prey patterns and the prevalence of katydids with particular defenses in the diet of gleaning bats, something that is not possible when the identification of prey remains is limited to the level of order or family.

When searching the literature for examples of katydid defenses against bats, we considered both physical defenses (such as large mandibles, chemical defenses, and large spines), and behavioral defenses (such as acoustic startle sounds, calling from protected perches, infrequent calling, vibratory tremulation signals, and song cessation in response to bat echolocation calls). The presence among the prey remains of two katydid species (*A. curvidens* and *C. brevirostris*) with large mandibles and a strong bite suggests that biting does not guarantee safety from substrate-gleaning bats, perhaps because many gleaners disable prey by biting the back of the katydid’s thorax, thus effectively evading its mandibles [28]. Chemical defenses are not well documented in katydids, but species in the genus *Vestria* are believed to produce a chemical deterrent when they are disturbed and it appears to be distasteful to monkeys [5]. No individuals of this genus were found in katydid remains at *M. hirsuta* roosts.

The presence of *Steirodon stalii* (Figure 1) in prey remains at bat roosts demonstrates that a large body size and the presence of thoracic spines do not make katydids impervious to gleaning bat predation. In a captive feeding study, *M. hirsuta* successfully caught and ate large katydids of the genus *Steirodon* (wings ca. 0.1 m, body ca. 0.065 m, weight ca. 4 g, compared to the 14 g *M. hirsuta*; [33,34]). However, we also noted that none of the katydids from Panama with exceptionally sharp and long spines (*Steirodon careovirgulatum, Markia hystrix, Stilpnochlora acanthonotum, Panacanthus spinosus*; [4]) were found in prey remains, suggesting that long, sharp spines could potentially deter bat predation. When observed within a flight cage, the gleaning bats *Trachops cirrhosus, Tonatia saurophila, Lophostoma silvicolum, M. microtis,* and *M. hirsuta* often subdue heavily armored katydids, but only after a considerable struggle and the bats occasionally end up with holes in their flight membranes (R. A. Page, pers. obs.). Interestingly, many gleaning bats that we have captured in mistnets on BCI have scars and holes in their wing and tail membranes, although these injuries can have many different causes.

Katydid species with known behavioral defenses were also found in the roost remains. Three katydid species (*Acanthodis curvidens*, *Pristonotus tuberosus,* and *Xestoptera cornea*), including one of the most commonly found species in the remains, are known to emit very loud sounds when grabbed by humans. These sounds are thought to function to startle predators [5]; however, whether the attack of a gleaning bat would trigger this behavior is unknown.

Much of the research on the interactions between katydids and substrate-gleaning bats has focused on the acoustic defenses of male katydids (e.g. use of sporadic/intermittent song). *Micronycteris hirsuta* are known to be attracted to prey-generated sounds like katydid mate-calling song [8,35] and will glean singing katydids while ignoring silent ones in captivity [35]. Some studies have shown that low duty cycle calls (duty cycle = call duration / signal period) or signaling with a low repetition rate reduces the probability of a predatory response by gleaning bats compared to high duty cycle calls or calling at a high repetition rate [8,26]. Many low duty cycle katydid species, however, were present in prey remains found at *M. hirsuta* roosts. Neotropical katydids with high duty cycles that sing continuously tend to call from restricted locations generally inaccessible to gleaning bats, such as thorny plants (*I. pulchripennis*) or tall grasses (*N. affinis*). Although we found remains of these two species in the bat cullings, they were either female or the sex could not be determined, so singing from protected locations could also be effective against gleaning bats. Different species of gleaning bats might exhibit differential preferences for exploiting prey-generated signals and thus exert different degrees of selection pressure on katydid sensory-based defenses [16,17]. In addition to passive acoustic defenses, such as low calling rates and calling from inaccessible locations, most katydids have acute ultrasonic hearing which allows them to detect the echolocation calls of passing bats and cease calling when they are in danger of an attack. Some katydids use this strategy, but others do not [22–24].

Perhaps the most striking result of our study is that silent female katydids were more commonly represented in the remains found in *M. hirsuta* roosts than male katydids that sing. We also found species of silent insects that are not known to emit acoustic mate-calling signals, including dragonflies, cockroaches, and beetles. The presence of many silent prey species and katydid species with acoustic-based defenses against gleaning bat predation suggest that eavesdropping on insect acoustic signals cannot be the only strategy used by *M. hirsuta* to find prey. Alternative strategies include eavesdropping on other kinds of incidental prey sounds, using echolocation to locate silent and motionless prey on vegetation, and catching prey in flight, also called aerial-hawking.

Several bat species are known to use incidental noises generated by insects to detect and locate them as prey, such as wing beat or flight sounds [36], landing and crawling sounds [37] and rustling noises [38,39]. Perhaps the general activity level of different prey species, including exposure time moving on various substrates, is an important risk factor governing gleaning bat predation. For example, during the day the katydid *D. gigliotosi* hides in plants on the forest floor and each night males make a long journey up into the canopy to sing and attract females [40], potentially producing a number of incidental locomotory noises along the way. A tracking study of *Phyllophilla ingens*, a medium-sized katydid common to our study area, revealed much higher levels of activity during the day than anticipated. Although the diurnal altitudinal movements of *P. ingens* included moving closer to the ground in the evening, just prior to the main activity period of predatory bats, they usually stayed ca. 10 m above ground [41]. Interestingly, *P. ingens* were not found in prey remains at *M. hirsuta* roosts even though they are readily captured and consumed by gleaning bats in captivity (33), and birds and rodents feed heavily upon them in the wild [41]. Little is known about the natural behavior of Neotropical katydids, hence additional research is needed to determine the behaviors that represent the greatest risk for exploitation by substrate-gleaning bats.

Another intriguing possibility is that *M. hirsuta* might use echolocation to find silent, motionless prey on surfaces, as has been demonstrated for the congeneric species *M. microtis* [27]. In a study investigating the responses of gleaning bats to katydid calls, *M. microtis* also showed the least interest in katydid calls compared to three other gleaning bat species in Panama [17], possibly because of greater reliance on echolocation for locating prey. Vegetation varies widely in its reflective properties [42]. For bats using echolocation cues to detect silent, motionless prey, the leaves that katydids perch on at night – either exposed or covered, smooth or textured, large or small – will vary in their echoacoustic properties and this likely changes the conspicuousness of substrate-borne insects to gleaning bats that use echolocation to find prey regardless of whether the prey actively emits sound. Therefore, habitat use by katydids is also an important risk factor for avoiding bats that detect prey on surfaces using echolocation.

A final possibility is that *M. hirsuta* are more flexible in foraging than previously believed and facultatively glean and catch prey in flight. Because a number of bat species have been shown to use both aerial-hawking and substrate-gleaning foraging strategies (e.g. *Cardioderma cor* [43], *Myotis auriculus* [44], *Nycteris grandis* and *N. thebaica* [45], *Hipposideros ruber* [46], *Megaderma lyra* [47], *R. ferrumequinum* and *R. hipposideros* [48], *M. emarginatus* [49], *M. evotis* [50], *M. lucifugus* and *M. septentrionalis* [51], *Rhinolophus blasii,* [52], *Otonycteris hemprichii* [53], *Megaderma spasma* [54]), this serves as a reminder to remain cautious about assigning bats to a single foraging strategy.

Flight cage experiments show that, in captivity, *M. hirsuta* can also both glean and catch insects in flight (33), meaning that not all prey in our study were necessarily captured by substrate-gleaning. A study on *M. spasma* in India showed that although these bats sometimes approached male katydid calling song, they always attacked tethered flying katydids [54]. In addition, the majority of katydid remains in the roosts of *M. spasma* were females, suggesting that the generally accepted idea that signaling males are at greater risk of predation than females does not hold true for katydids in this system [54]. Our data support this conclusion for Neotropical katydids as well. Some katydid species are known to have a diving response to ultrasound in flight [55,56], as do crickets [57] and some beetles [58,59]. It is interesting to note, however, that the majority of katydid remains in the roosts of *M. hirsuta* were from species in the subfamily Pseudophyllinae, which are not strong fliers (Lang and Römer, pers. obs.). In addition, there were a number of remains of dragonflies (Odonata), which are not active at night. Therefore, it is likely that *M. hirsuta* is using a variety of strategies to catch prey.

Taken together, our data indicate that the predator–prey relationship between Neotropical gleaning bats and katydids is more complicated than previously thought. Some katydid defenses, such as exceptionally long spines and calling from dense vegetation, might be successful in reducing the probability of being captured by some Neotropical gleaning bats, whereas others, such as large mandibles, sporadic calling, or even silence, do not guarantee safety. In addition, the large number of silent prey in the diet of *M. hirsuta*, including a very large proportion of female katydids, means that these bats must be using a variety of strategies to locate prey. More research is needed on the interactions and behavior of these animals to assess the many factors that potentially contribute to this predator–prey system.

## Author contributions

DKND and SLV-H collected insect remains; HtH classified katydid wings to sex and side; all authors contributed to the analysis and writing of the manuscript.

*Associate Editor:* Nathan Muchhala

## Disclosure statement

No potential conflict of interest was reported by the authors.

## Funding

Funding was provided by a STRI Short-Term Fellowship and Dartmouth College to HtH, the Austrian Science Fund (FWF; Projects P14257 and P17986-B06) to HR, STRI and Human Frontier Science Program [grant number RGP0040/2013] to RAP, the Natural Sciences and Engineering Research Council (NSERC) of Canada to PAF, and the Roche Research Foundation and the ZUNIV-Fonds zur Förderung des Akademischen Nachwuchses (FAN) to DKND.
